# Disease progression in proposed brain-first and body-first Parkinson’s disease subtypes

**DOI:** 10.1038/s41531-024-00730-1

**Published:** 2024-06-04

**Authors:** Zhiheng Xu, Tianyu Hu, Chenqin Xu, Xiaoniu Liang, Shiyu Li, Yimin Sun, Fengtao Liu, Jian Wang, Yilin Tang

**Affiliations:** Department of Neurology and National Research Center for Aging and Medicine and National Center for Neurological Disorders, State Key Laboratory of Medical Neurobiology, Huashan Hospital, Fudan University, Shanghai, China

**Keywords:** Parkinson's disease, Medical research

## Abstract

A new Parkinson’s disease (PD) subtyping model has been recently proposed based on the initial location of α-synuclein inclusions, which divides PD patients into the brain-first subtype and the body-first subtype. Premotor RBD has proven to be a predictive marker of the body-first subtype. We found compared to PD patients without possible RBD (PD^pRBD–^, representing the brain-first subtype), PD patients with possible premotor RBD (PD^pRBD+^, representing the body-first subtype) had lower Movement Disorders Society Unified Parkinson’s Disease Rating Scale part III (MDS UPDRS-III) score (*p* = 0.022) at baseline but presented a faster progression rate (*p* = 0.009) in MDS UPDRS-III score longitudinally. The above finding indicates the body-first subtype exhibited a faster disease progression in motor impairments compared to the brain-first subtype and further validates the proposed subtyping model.

## Introduction

Parkinson’s disease (PD) is a heterogeneous neurodegenerative disorder exhibiting a wide range of phenotypes^1^. This diversity has prompted the proposal of different PD subtypes, aiming to improve our understanding of the underlying disease mechanisms and provide personalized management. However, despite these efforts, a unified and widely accepted classification system for PD subtypes is still lacking, and the pathophysiological foundations of PD heterogeneity remain elusive.

To fully classify patients and account for the underlying pathophysiological changes, one intriguing hypothesis was recently proposed: the brain-first subtype and body-first subtype^2^. For the past two decades, the Braak’s staging system^3^ has been a dominant hypothesis, which postulated that α-synuclein, the key neuropathological feature of PD, originates in the dorsal motor nucleus of the vagus. However, robust evidence now indicates that the Braak staging system is not valid for all cases at post mortem and pathology might start in other brain regions in a subset of patients^4–6^.

To address this controversy, the brain-first and body-first models were introduced. According to the theory, PD can be divided into two distinct subtypes based on the initial site of α-synuclein pathology and its spreading pattern^7^. In the brain-first subtype, α-synuclein pathology initially arises in the brain and subsequently spreads to the peripheral autonomic nervous system. Conversely, in the body-first subtype, the pathology originates in the enteric or peripheral autonomic nervous system and then spreads to the brain. Notably, the presence of rapid eye movement sleep behavior disorder (RBD) may be indicative of a body-first pattern^7,8^, as propagating body-first pathology will affect the pons before reaching the substantia nigra^9^.

Previous studies have verified the brain-first and body-first subtypes cross-sectionally, using both clinical multimodal imaging techniques^7,8^ and postmortem pathological findings^4–6^, which support the patient stratification based on the brain-first/body-first spreading hypothesis. However, to our knowledge, longitudinal study of the above subtypes is still lacking and little is known about the disease progression of the proposed brain-first and body-first subtypes. Therefore, we aimed to analyze the longitudinal changes of motor and non-motor features of the proposed brain-first and body-first subtypes, thus further validating the proposed subtyping model.

## Results

We enrolled a total of 137 PD patients in this study, 64 patients in the patients without possible RBD group (PD^pRBD–^ group, baseline RBDSQ ≤ 3, baseline RBDSQ: 1.72 ± 0.93) and 73 patients in the patients with possible premotor RBD group (PD^pRBD+^ group, baseline RBDSQ ≥ 6, baseline RBDSQ: 8.45 ± 2.02) respectively. Sex distribution was statistically different between two groups (*p* = 0.007, Table 1). Compared to the PD^pRBD–^ group, the PD^pRBD+^ group had lower Movement Disorders Society Unified Parkinson’s Disease Rating Scale part III (MDS UPDRS-III) score (*p* = 0.022) and higher Non-motor Symptoms Scale (NMSS) score (*p* = 0.001) at baseline (Table 1), indicating the body-first subtype presented fewer motor impairments but more nonmotor impairments compared to the brain-first subtype. No statistical differences were found in Epworth Sleepiness Scale (ESS) score, Mini Mental State Examination (MMSE) score, Beck Depression Inventory (BDI) score, Parkinson Disease Questionnaire 39 (PDQ-39) score and levodopa equivalent daily dose (LEDD) between two groups. Subscores of MDS UPDRS-III and NMSS by group are listed in the Supplementary Table 1.

**Table 1:** Demographic profiles, baseline clinical characteristics and estimates for change in clinical scores of enrolled patients by group

Variables	PD^pRBD–^ (*n* = 64)	PD^pRBD+^ (*n* = 73)	*p* value
*Basic Demographic Profiles*			
Age (years)	63.42 (6.68)	61.29 (10.89)	0.921
Sex (female)	32 (50.00%)	20 (27.39%)	**0.007**
Education (years)	11.73 (4.19)	11.88 (3.69)	0.828
Disease duration (months)	65.44 (50.21)	63.74 (60.00)	0.245
Age of onset (years)	58.13 (6.85)	55.99 (11.98)	0.769
*Baseline Clinical Characteristics*			
MDS UPDRS-III score (med-off)	29.63 (10.57)	24.69 (13.74)	**0.022**
NMSS score	9.15 (4.68)	12.29 (5.28)	**0.001**
ESS score	6.10 (3.54)	6.46 (4.60)	0.876
MMSE score	27.60 (2.15)	27.39 (2.80)	0.998
BDI score	10.87 (6.32)	14.03 (9.68)	0.089
PDQ-39 score	25.10 (18.16)	33.86 (25.65)	0.093
LEDD	346.35 (208.02)	460.52 (532.39)	0.600
*Estimates for Change in Clinical Scores*			
MDS UPDRS-III score (med-off)	0.071 (0.023)	0.150 (0.019)	**0.009**
NMSS score	0.090 (0.009)	0.083 (0.008)	0.729
ESS score	0.020 (0.008)	0.029 (0.007)	0.362
MMSE score	-0.020 (0.007)	-0.017 (0.006)	0.767
BDI score	0.041 (0.015)	0.054 (0.017)	0.637
PDQ-39 score	0.229 (0.041)	0.291 (0.039)	0.278

Basic Demographic Profiles and Baseline Clinical Characteristics: Data are mean (SD) or n (%). Two-tailed p values are presented, and differences were considered statistically significant at *p* < 0.05 (highlighted in bold). Chi-squared test was used for comparing sex distribution. Mann–Whitney U test was used for comparing age, education, disease duration, age of onset, NMSS score, ESS score, MMSE score, BDI score, PDQ-39 score, and LEDD. Student *t* test was used for comparing MDS UPDRS-III score. Estimates for Change in Clinical Scores: Data are estimate β (SD). For MDS UPDRS-III score, NMSS score, and ESS score, the analyses were corrected for gender, age at baseline, and LEDD; while for MMSE score, BDI score, and PDQ-39 score, gender, age at baseline, LEDD, and years of education were corrected.

*PD*^*pRBD*–^ PD patients without possible RBD, *PD*^*pRBD+*^ PD patients with possible premotor RBD, *BDI* Beck Depression Inventory, *ESS* Epworth Sleepiness Scale, *LEDD* levodopa equivalent daily dosage, *MMSE* Mini Mental State Examination, *NMSS* non-motor symptoms scale, *PDQ-39* Parkinson Disease Questionnaire 39, *MDS UPDRS-III* Movement Disorders Society Unified Parkinson’s Disease Rating Scale part III.

**Fig. 1:**
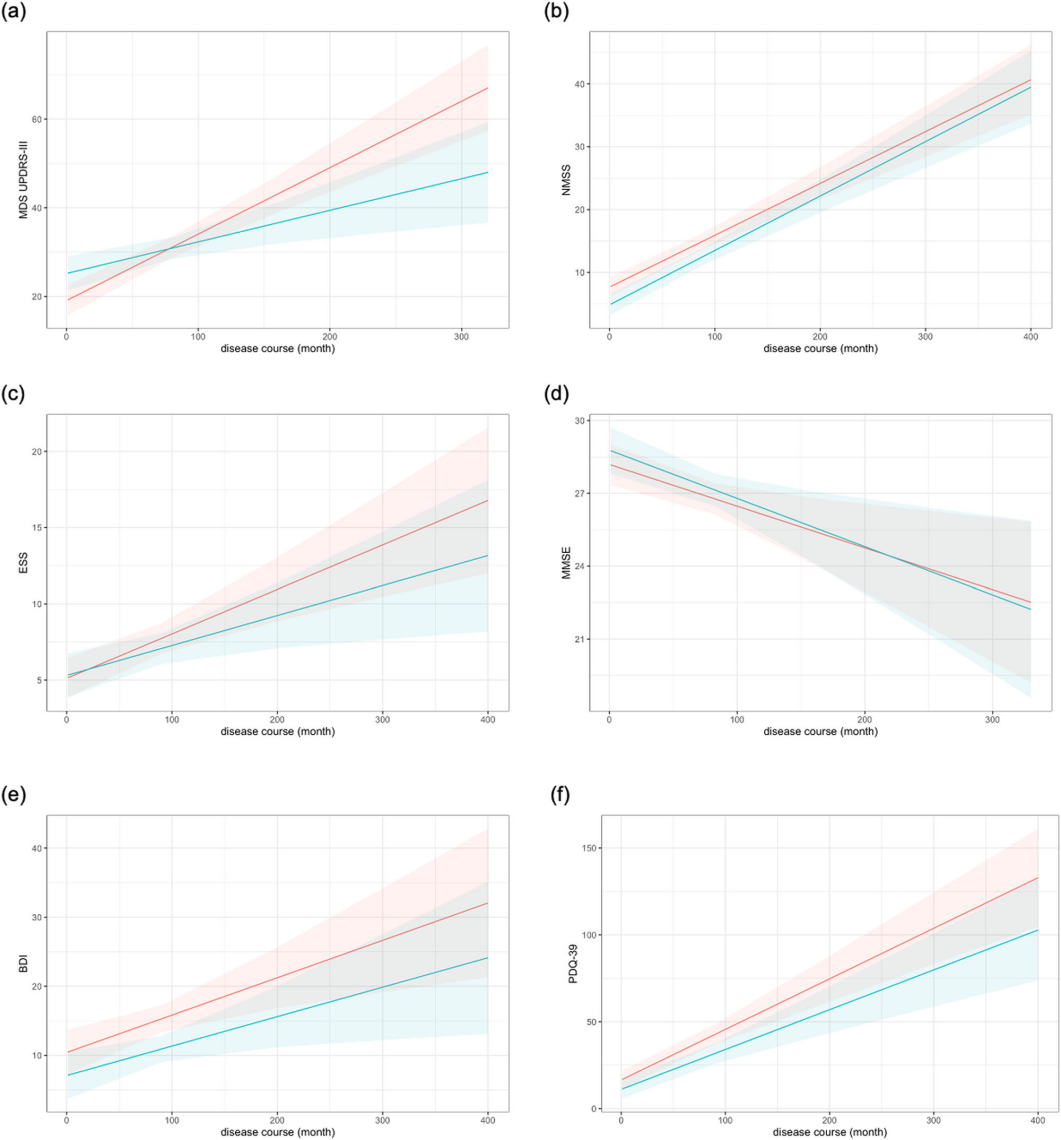
Longitudinal trajectories of clinical characteristics by group. Linear mixed models indicate that MDS UPDRS-III score trajectories over time are statistically different between two groups, with the PD^pRBD+^ group (red line, the body-first subtype), worsening at a faster rate than the PD^RBD-^ group (blue line, the brain-first subtype) (**a**). No statistical differences were found in disease progression rates regarding NMSS score (**b**), ESS score (**c**), MMSE score (**d**), BDI score (**e**) and PDQ-39 score (**f**) between two groups. Abbreviations: PD^pRBD–^, PD patients without possible RBD; PD^pRBD+^, PD patients with possible premotor RBD. BDI Beck Depression Inventory; ESS Epworth Sleepiness Scale; LEDD levodopa equivalent daily dosage; MMSE Mini Mental State Examination; NMSS non-motor symptoms scale; PDQ-39 Parkinson Disease Questionnaire 39; MDS UPDRS-III Movement Disorders Society Unified Parkinson’s Disease Rating Scale part III.

Linear mixed models, adjusting for gender, age at baseline, and LEDD, were used to analyze the difference in the MDS UPDRS-III score, NMSS score, and ESS score between the PD ^pRBD–^ group and PD^pRBD+^ group. For MMSE score, BDI score, and PDQ-39 score, LEDD was further adjusted. We found the rate of change in MDS UPDRS-III score per month reached statistical significance between two groups (*p* = 0.009), with PD^pRBD+^ group, the body-first subtype, worsening at a statistically faster rate (PD^pRBD+^ group estimate *β* = 0.150 ± 0.019, PD^pRBD-^ group estimate *β* = 0.071 ± 0.023) (Table 1, Fig. 1a). No statistical differences were found in disease progression rates regarding NMSS score (Table 1, Fig. 1b), ESS score (Table 1, Fig. 1c), MMSE score (Table 1, Fig. 1d), BDI score (Table 1, Fig. 1e) and PDQ-39 score (Table 1, Fig. 1f) between two groups.

## Discussion

The above results revealed notable differences between the PD^pRBD+^ group and PD^pRBD–^ group, suggesting body-first subtype exhibited fewer motor impairments (lower MDS UPDRS-III score) and more nonmotor impairments (higher NMSS score) compared to the brain-first subtype at baseline and a much faster disease progression in motor impairments, manifested by rapid increase in MDS UPDRS-III score.

Firstly, we have identified a notable sex distribution difference between two groups, as PD^pRBD+^ group presented a higher portion of male patients. The observed sex distribution discrepancy is consistent with previous findings, which found male were at higher risk of confirmed RBD in clinical populations than female^10^ and presented significantly more aggressive behavior during dreams^11,12^. This sex-based difference warrants further exploration in larger cohorts to ascertain its reproducibility and potential implications for clinical management.

Furthermore, our comparative analysis of motor impairments between the two groups has unveiled intriguing patterns. The PD^pRBD+^ group, representing the body-first subtype, exhibited lower MDS UPDRS-III score compared to the PD^RBD-^ group, the brain-first subtype, at baseline. More importantly, the longitudinal assessment of disease progression revealed a statistically significant difference in the rate of change in MDS UPDRS-III score between two groups as PD^pRBD+^ group presented a much faster disease progression in motor impairments. The severity of impairments has long been linked to the pathological burden of α-synuclein^13^. In the body-first subtype, the pathology originates in the enteric or peripheral autonomic nervous system and then spreads to the brain. It has been suggested that α-synuclein may traverse multiple synapses to reach the substantia nigra, thereby causing motor impairments. In light of our baseline finding, we speculate that the body-first subtype may present fewer motor impairments in the initial stages due to less substantia nigra involvement. Borghammer^13^ has hypothesized that as the disease advances, the global burden of α-synuclein pathology is higher in the body-first subtype due to the more symmetric involvement of both hemispheres, possibly further promoted by the more marked involvement of ascending, neuromodulatory brainstem nuclei, leading to an elevated risk of faster progression. This hypothesis has not been validated in longitudinal cohort before, and our study here might provide a support for this concept, which needs further validation.

Additionally, we also found PD^pRBD+^ group exhibited higher NMSS score at baseline. This aligns with the notion that body-first patients should theoretically show damage to the autonomic nervous system and the lower brainstem structures earlier in the disease course compared to brain-first patients^4^ and is supported by the previous finding that body-first subtype is associated with non-motor dominant phenotype^14^. Therefore, our study adds to the prior body of evidence that the body-first subtype shows more severe non-motor impairments than the brain-first subtype.

Several limitations should be acknowledged. Firstly, the sample size in our exploratory study may limit the generalizability of the findings. Future research with larger cohorts is warranted to confirm and expand upon these results. Moreover, RBD was assessed by an accessible screening questionnaire RBDSQ. Though RBDSQ exhibits good diagnostic accuracy in the general population^15^ and we have classified patients using stringent thresholds, the inclusion criteria in this study was still not identical to the gold standard polysomnography. Additionally, the brain-first and body-first PD subtypes are most strongly distinguished early in the disease course and RBD disease state could have mitigated some difference between two groups. And recall bias may present due to long disease duration. Lastly, this study focused on a specific set of clinical features; further investigations incorporating imaging or biomarker data may provide a more comprehensive understanding of the underlying mechanisms driving subtype-specific progression.

In conclusion, our preliminary exploratory study contributes to the growing body of literature of the proposed brain-first and body-first subtypes and explores the longitudinal disease progression by subtypes. We have found that compared to the brain-first subtype, the body-first subtype exhibited a much faster disease progression in motor impairments, manifested by rapid increase in MDS UPDRS-III score. Future research should further validate the proposed subtyping model and delve deeper into the neurobiological underpinnings of these subtypes to inform targeted interventions and enhance the precision of PD management.

## Methods

### Patients and grouping

PD patients were enrolled from the Department of Neurology, Huashan Hospital, Fudan University. PD diagnosis for each patient was determined by three movement disorder specialists according to the UK PD Society Brain Bank Clinical Diagnostic Criteria^16^ (for patients recruited before 2016) and the 2015 MDS Clinical Diagnostic Criteria for PD^17^ (for patients recruited from 2016 on). The Human Studies Institutional Review Board, Huashan Hospital, Fudan University approved this study. Informed consents were obtained.

Despite polysomnography (PSG) is widely recognized as the gold standard for diagnosing RBD^18^, REM Sleep Behavior Disorder Screening Questionnaire (RBDSQ) was used due to the limited availability of polysomnography data, which exhibits good diagnostic accuracy in the general population^15^. To avoid the uncertainty caused by scores, we include classified patients without possible RBD (PD^pRBD–^ group) as scoring ≤3 and patients with possible premotor RBD (PD^pRBD+^ group) as scoring ≥6 at baseline, same as one previous study defined^8^. 28 PD patients, who scored 4 or 5 on RBDSQ at baseline, were excluded. Their demographic profiles, baseline clinical characteristics, and disease progression rates were listed in the Supplementary Table 2 and Supplementary Table 3. For PD^pRBD+^ patients, subjective RBD sleep symptoms needed to manifest before the onset of motor symptoms. Thus, according to the above inclusion criteria, a total of 137 PD patients were enrolled in this study, 64 patients in PD^pRBD–^ group and 73 patients in PD^pRBD+^ group respectively. The average follow-up time were 65.84 ± 32.55 months (maximum follow-up time: 124 months) in the PD^pRBD–^ group and 60.75 ± 32.65 (maximum follow-up time: 133 months) in the PD^pRBD+^ group.

### Clinical assessments

The standardized procedure detailed here was performed through a face-to-face interview with all PD patients. An established method was used to calculate the levodopa equivalent daily dose (LEDD)^19^. The Movement Disorders Society Unified Parkinson’s Disease Rating Scale part III (MDS UPDRS-III) were conducted during the off-medication state, defined as the withdrawal of anti-PD medications for at least 12 h, except in those who could not tolerate it. Non-motor Symptoms Scale (NMSS), Epworth Sleepiness Scale (ESS), Mini Mental State Examination (MMSE), Beck Depression Inventory (BDI), and Parkinson Disease Questionnaire 39 (PDQ-39) were also conducted. All patients were scheduled for an annual follow-up clinical assessment in the same month (±2 weeks) as in the previous year in our institution, if they were available.

### Statistical analysis

To compare the baseline demographic profiles and clinical characteristics between the two groups, Chi-squared test was used for categorical variables and student *t* test and Mann–Whitney U test were used for continuous variables as appropriate. To compare the longitudinal changes in clinical characteristics between the two groups, a linear mixed-effect model was applied. Disease duration was used as the time scale. A participant-specific random effect was considered due to the correlations among repeated measurements within the same participants. For MDS UPDRS-III score, NMSS score, and ESS score, the analyses were corrected for gender, age at baseline and LEDD; while for MMSE score, BDI score, and PDQ-39 score, gender, age at baseline, LEDD, and years of education were corrected. Two-tailed *p* values are presented, and differences were considered statistically significant at *p* < 0.05.

### Supplementary information

Supplementary Data

## Data Availability

Due to privacy concerns, the original data (demographic information, questionnaires) cannot be publicly disclosed. However, the cleaned clinical data are available from the corresponding author (Y.T., J.W.) via mail.
