# Supplementary material for: Disease progression in proposed brain-first and body-first Parkinson’s disease subtypes
Source: NPJ Parkinsons Dis. 2024 Jun 4;10:111. doi: 10.1038/s41531-024-00730-1 (PMC11150376; doi:10.1038/s41531-024-00730-1)
Supplement: Supplementary file 1 — Supplementary Data [file 41531_2024_730_MOESM1_ESM.pdf]

Supplementary Table 1. Subscores of MDS UPDRS-III and NMSS of enrolled patients by group at baseline

Variables	PD^{pRBD-} (n=64)	PD^{pRBD+} (n=73)	p-value
Subscores of MDS UPDRS-III			
bradykinesia subscore	11.59 (4.80)	8.89 (5.94)	**0.002**
tremor subscore	3.77 (2.79)	3.22 (3.69)	**0.048**
rigidity subscore	5.50 (3.43)	4.81 (3.82)	0.179
posture and gait disorder subscore	4.14 (2.27)	3.65 (2.60)	0.183
Subscores of NMSS			
domain 1: cardiovascular including falls	0.81 (0.65)	0.75 (0.72)	0.564
domain 2: sleep/fatigue	0.97 (1.02)	0.99 (0.95)	0.757
domain 3: mood /cognition	2.31 (1.41)	2.82 (1.49)	**0.034**
domain 4: perceptual problems/ hallucinations	0.66 (0.90)	1.08 (0.91)	**0.005**
domain 5: attention/memory	1.00 (1.07)	1.30 (1.14)	0.124
domain 6: gastrointestinal tract	0.73 (0.79)	1.10 (0.85)	**0.010**
domain 7: urinary	1.16 (0.89)	1.58 (0.90)	**0.008**
domain 8: sexual function	0.63 (0.71)	1.37 (0.70)	**<0.001**
domain 9: miscellaneous	0.94 (0.94)	1.26 (1.05)	0.072

Data are mean (SD) or n (%). Two-tailed p values are presented, and differences were considered statistically significant at $p<0.05$. Mann–Whitney U test was used for comparison. Abbreviations: PD^{pRBD-}, PD patients without possible RBD; PD^{pRBD+}, PD patients with possible premotor RBD. NMSS, non-motor symptoms scale; MDS UPDRS-III, Movement Disorders Society Unified Parkinson’s Disease Rating Scale part III.

Supplementary Table 2. Demographic profiles and baseline clinical characteristics of enrolled patients by RBD scores

Variables	PD^{pRBD-} (n=64)	PD^{pRBDs} (n=28)	PD^{pRBD+} (n=73)	p-value
Basic Demographic Profiles				
Age (years)	63.42 (6.68)	59.46 (8.32)	61.29 (10.89)	0.238
Sex (female)	32 (50.00%)	14 (50.00%)	20 (27.39%)	**0.013**
Education (years)	11.73 (4.19)	9.90 (4.39)	11.88 (3.69)	0.070
Disease duration (months)	65.44 (50.21)	69.50 (42.07)	63.74 (60.00)	0.248
Age of onset (years)	58.13 (6.85)	53.29 (8.01)	55.99 (11.98)	0.063
Baseline Clinical Characteristics				
MDS UPDRS-III score (med-off)	29.63 (10.57)	34.29 (14.90)	24.69 (13.74)	**0.003**
NMSS score	9.15 (4.68)	11.75(4.59)	12.29 (5.28)	**0.002**
ESS score	6.10 (3.54)	5.57 (3.93)	6.46 (4.60)	0.663
MMSE score	27.60 (2.15)	27.86 (2.10)	27.39 (2.80)	0.744
BDI score	10.87 (6.32)	12.43 (7.63)	14.03 (9.68)	0.216
PDQ-39 score	25.10 (18.16)	38.07 (26.66)	33.86 (25.65)	0.083

Basic Demographic Profiles and Baseline Clinical Characteristics: Data are mean (SD) or n (%). Two-tailed p values are presented, and differences were considered statistically significant at $p<0.05$. Chi-squared test was used for comparing sex distribution. Kruskal--Wallis H test was used for comparing age, education, disease duration, age of onset, MDS UPDRS-III score, NMSS score, ESS score, MMSE score, BDI score, and PDQ-39 score.

Abbreviations: PD^{pRBD-}, PD patients without possible RBD (RBDSQ⩽3 at baseline); PD^{pRBDs}, PD patients with possible premotor RBD suspected (RBDSQ =4 or 5 at baseline); PD^{pRBD+}, PD patients with possible premotor RBD (RBDSQ⩾6 at baseline). BDI, Beck Depression Inventory; ESS, Epworth Sleepiness Scale; LEDD, levodopa equivalent daily dosage; MMSE, Mini Mental State Examination; NMSS, non-motor symptoms scale; PDQ-39, Parkinson Disease Questionnaire 39; MDS UPDRS-III, Movement Disorders Society Unified Parkinson's Disease Rating Scale part III. Student t test was used for comparing MDS UPDRS-III score.

Supplementary Table 3. Estimates for change in clinical scores of enrolled patients by RBD scores

**Variables**	**PD^{pRBD-} (n=64)**	**PD^{pRBDs} (n=28)**	**PD^{pRBD+} (n=73)**	**PD^{pRBD-} vs PD^{pRBDs} p-value**	**PD^{pRBD-} vs PD^{pRBD+} p-value**	**PD^{pRBDs} vs PD^{pRBD+} p-value**
Estimates for Change in Clinical Scores						
MDS UPDRS-III score (med-off)	0.071 (0.023)	0.031 (0.037)	0.150 (0.019)	0.353	**0.009**	**0.007**
NMSS score	0.090 (0.009)	0.052 (0.017)	0.083 (0.008)	0.069	0.729	0.121
ESS score	0.020 (0.008)	0.005 (0.013)	0.029 (0.007)	0.319	0.362	0.116
MMSE score	-0.020 (0.007)	-0.007 (0.011)	-0.017 (0.006)	0.341	0.767	0.428
BDI score	0.041 (0.015)	0.048 (0.032)	0.054 (0.017)	0.846	0.637	0.865
PDQ-39 score	0.229 (0.041)	0.228 (0.070)	0.291 (0.039)	0.973	0.278	0.429

Estimates for Change in Clinical Scores: Data are estimate β (SD). For MDS UPDRS-III score, NMSS score and ESS score, the analyses were corrected for gender, age at baseline and LEDD; while for MMSE score, BDI score, and PDQ-39 score, gender, age at baseline, LEDD and years of education were corrected.

Abbreviations: PD^{pRBD-}, PD patients without possible RBD (RBDSQ⩽3 at baseline); PD^{pRBDs}, PD patients with possible premotor RBD suspected (RBDSQ =4 or 5 at baseline); PD^{pRBD+}, PD patients with possible premotor RBD (RBDSQ⩾6 at baseline). BDI, Beck Depression Inventory; ESS, Epworth Sleepiness Scale; LEDD, levodopa equivalent daily dosage; MMSE, Mini Mental State Examination; NMSS, non-motor symptoms scale; PDQ-39, Parkinson Disease Questionnaire 39; MDS UPDRS-III, Movement Disorders Society Unified Parkinson's Disease Rating Scale part III. Student t test was used for comparing MDS UPDRS-III score.
